# Clinical Performance of a Liquid Preservation Medium for Cervicovaginal Samples in DNA‐HPV Testing and Liquid‐Based Cytology for Cervical Cancer Screening

**DOI:** 10.1111/cyt.13495

**Published:** 2025-04-23

**Authors:** Larissa Dias Assunção, Michelle Garcia Discacciati, Andressa Germano da Silva, Adriana Yoshida, Diama Bhadra Vale, Julio Cesar Teixeira

**Affiliations:** ^1^ Department of Obstetrics and Gynecology University of Campinas (UNICAMP) Campinas São Paulo Brazil; ^2^ Department of Pathology Federal University of Sao Paulo Sao Paulo Brazil

**Keywords:** cancer screening, cervix cancer, cytological techniques, DNA‐HPV test, human papillomavirus, Pap smear

## Abstract

**Introduction:**

Cervical cancer remains a significant global health concern, primarily associated with persistent infections by high‐risk human papillomavirus (hr‐HPV). As screening programmes evolve from traditional cytology to DNA‐HPV testing, the need for a liquid medium that maintains the integrity of cervical samples for biomolecular analysis and cytology becomes critical.

**Methods:**

This study evaluated the performance of the candidate liquid preservation medium (PM) Cytoliq for cervical samples intended for DNA‐HPV testing and liquid‐based cytology (LBC), in comparison with the reference PM, PreservCyt‐ThinPrep. A total of 112 women aged 18–64 years underwent routine gynaecological examinations, with paired cervical samples preserved in both PM for HPV testing and genotyping (Cobas HPV test), and LBC. The study aimed for a sensitivity greater than 90% in detecting cervical intraepithelial neoplasia grade 2 or worse (CIN2+), moderate to high agreement in HPV testing results (Kappa index > 0.70) and adequate performance in LBC.

**Results:**

The candidate PM exhibited non‐inferior performance relative to the reference PM. DNA‐HPV testing showed a 94.5% agreement rate (Kappa = 0.88) and a sensitivity of 92.9% for CIN2+ detection. Additionally, the candidate PM performed well in LBC smear production, with no significant differences in cytological diagnoses. The agreement in LBC diagnoses was 94.0% (Kappa = 0.79) with the ThinPrep processor and 91.8% (Kappa = 0.63) with the Cytoliq processor.

**Conclusion:**

The Cytoliq PM demonstrated comparable efficacy to the reference for DNA‐HPV testing and LBC, supporting its potential as an alternative preservation medium in cervical cancer screening programmes.

## Graphical abstract

The study evaluated the performance of a new liquid preservation medium for cervical samples in DNA‐HPV testing and liquid‐based cytology. The candidate medium demonstrated a 94.5% agreement rate in HPV testing, 92.9% sensitivity for detecting CIN2+ and good performance in liquid‐based cytology. The findings support its potential as a viable alternative for cervical cancer screening.

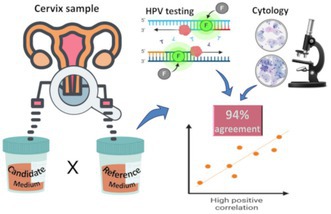

## 1 Introduction

Cervical cancer is a significant global health issue. In Brazil, it is estimated that there will be 17,010 new cases and 6700 deaths annually from 2023 to 2025, making it the third most prevalent cancer and the fourth leading cause of cancer‐related mortality in women [1, 2]. This cancer is associated with persistent infection by a high‐risk oncogenic human papillomavirus (hr‐HPV), which leads to the development of precursor lesions and, after several years, the emergence of carcinoma [3]. This progression pattern allows for screening at‐risk women through periodic testing of cervical samples [3].

The first generation of screening programmes began in the 1970s with conventional cytology or the Pap test, which is still used in many countries [4, 5, 6]. In regions where these programmes have been well organised and maintained, with high coverage of the target population, an 80% reduction in mortality has been demonstrated [4, 5, 6]. Later, the identification of HPV as the cause of cervical cancer and its potential detection through biomolecular tests led to several studies demonstrating clear superiority in identifying at‐risk women using DNA‐HPV detection in cervical samples [7, 8, 9]. With DNA‐HPV tests based on polymerase chain reaction (PCR) technology, there was an increase in sensitivity and reproducibility, generating confidence, extending the retesting interval and enabling earlier detection of precursor lesions or early‐stage cancer by several years [7, 8, 9, 10]. This superiority is even more apparent in low‐prevalence settings, such as in populations previously vaccinated against HPV [11], which will gradually dominate in many settings.

Thus, since 2017, some countries have begun transitioning their programmes from cytology‐based methods to the primary use of DNA‐HPV testing [12, 13, 14]. In this context, based on the DNA‐HPV test result, liquid‐based cytology (LBC) is used as a triage for colposcopy in about 10% of cases performed on the same sample (reflex cytology) [10, 14]. Introduced in the early 2000s, the LBC processor uses automated technology that includes cell homogenisation, debris filtration and transferring cells to slides in a thin layer, resulting in more uniform and clear slides with a lower rate of unsatisfactory samples [15, 16]. Using a liquid medium that preserves sample integrity and allows for biomolecular testing and to achieve adequate LBC is crucial.

In Brazil, after decades of opportunistic cytology programmes with no impact on the high mortality caused by cervical cancer, a transition to an organised programme based on DNA‐HPV testing began in 2024 [17]. The current scenario predicts a high demand for tests and supplies, and the development of alternatives could assist in implementing the programme on a national scale at an affordable cost. This study evaluated the clinical performance of a cell preservation solution as a candidate liquid medium for collecting and preserving cervicovaginal samples for screening, DNA‐HPV detection and LBC processing.

## 2 Methods

A diagnostic test study was conducted to assess the performance of the candidate liquid preservation medium (PM) Cytoliq (Erviegas Group, Indaiatuba, SP, Brazil) in preserving cervicovaginal samples for DNA‐HPV testing and LBC, compared to the performance of the reference medium PreservCyt‐Thinprep (Hologic Inc., Marlborough, MA, United States). The candidate PM was developed in Brazil for general cytology and contains ethyl alcohol, methylparaben and sodium chloride, with biochemical evaluation that led to its registration by the Brazilian Health Regulatory Agency (ANVISA) (registration no. 10039370007, 1/JUL/2021) [18].

Guidelines from an international HPV test validation manual [19], the cornerstone of current screening programmes, were used as a reference. The guideline recommends the HPV testing needs to achieve a sensitivity greater than 90% in detecting cervical intraepithelial neoplasia grade 2 or worse (CIN2+), including cervical cancer, and a high agreement in test results (Kappa index > 0.70). In this study, we used these criteria to compare the test results of samples preserved in the candidate PM with those preserved in the reference PM. The sample size was calculated assuming similar sensitivities between the PMs and moderate agreement, resulting in a required sample size of 100 samples per PM with a statistical power of 99% [19].

The study evaluated 116 women aged 25–64 years who attended routine gynaecological evaluations or follow‐up after conventional cytology or treatment for precursor lesions at the Women's Hospital of the University of Campinas, Campinas (SP), Brazil, from June to December 2023. Four women were excluded due to one of the following conditions: recent use of vaginal medications or immunosuppressive therapies, menstrual bleeding or history of pelvic radiotherapy. Thus, 112 women were included, and all signed an informed consent form. During the gynaecological speculum examination and after the routine cervical cytology sample collection, two additional samples were collected and placed in two vials containing the candidate and reference PM. The collection order was randomised in a 1:1 ratio for the first collection, and the tubes were labelled with codes to prevent assessors from knowing the case details or sample pairing. The code was revealed only for statistical analysis. The DNA‐HPV test used was the Cobas HPV test (Roche Molecular Systems, Pleasanton, CA, United States), which detects genotypes 16, 18 and 12 other hr‐HPV types in a grouped format. The agreement between the molecular test results was evaluated using the Kappa index and interpreted according to Landis and Koch [20]: < 0.1 = absent; 0.10 to 0.20 = slight; 0.21 to 0.40 = fair; 0.41 to 0.60 = moderate; 0.61 to 0.80 = substantial; and 0.81 to 1 = excellent. According to the HPV test validation guideline, the target Kappa value to achieve is > 0.7, indicating high agreement [19].

The LBC slides were prepared automatically using equipment from the same manufacturers of the preservation mediums, namely the Cytoliq LBC processor (Erviegas Group, Indaiatuba, SP, BR) and the Thinprep TP2000 processor (Hologic Inc., Marlborough, MA, United States). Two LBC slides were produced from each preserved sample, one on each processor, allowing for the assessment of the interchangeability of the two PM across the two processors. Subsequently, the slides were scanned using the KFBIO KF‐PRO‐400 scanner (Konfoong Bioinformation Tech Co., Ningbo, Zhejiang, CN). Cytological analyses followed the Bethesda System criteria for cellularity quality, presence of the cervix's squamocolumnar cell junction (SCJ), presence of obscuring factors, sample adequacy and cytological diagnosis [21, 22]. Two independent and experienced cytologists read the LBC slides, and any discordant results were reviewed until consensus. The Kappa index and descriptive analysis evaluated the agreement between results by PM and LBC processors. Statistical analysis was performed using the StatDirect 3.0 software (Wirral, United Kingdom, www.statsdirect.com). The Ethics Committee of the University of Campinas approved the study.

## 3 Results

The 112 women included in the study were aged between 25 and 64 (mean age: 36.8 years). Of these, 76 were undergoing screening, 24 were following up after an abnormal screening with conventional cytology and 12 were in post‐treatment follow‐up. The first sample collection occurred in 59 cases for the candidate PM and 53 cases for the reference PM (*p* = 0.423). Three cases had insufficient samples for DNA extraction: one case for both mediums and two cases for the reference PM only, both as the first collection. Therefore, DNA‐HPV testing was performed in 99.1% (111/112) samples preserved in the candidate PM, which was not inferior to the 97.3% (109/112) tested for the reference PM (*p* = 0.311). The 109 paired cases for both PM showed the same proportion of hr‐HPV detection, 34.9% (38/109). The results by the medium group were concordant in 94.5% (106/109), with a Kappa index of 0.88 (excellent agreement). Considering genotyping, there was one case with positive tests for both HPV types 16 and 18, concordant in both mediums and counted as two individual results. Thus, the concordance considering genotyping was 94.4% (102/110), with a Kappa index of 0.89, indicating almost perfect agreement (Table 1).

**TABLE 1:** Agreement between different hr‐HPV test results in cervical samples by preservation medium.

hr‐HPV testing in the reference medium^a^	hr‐HPV testing in the candidate medium (*n*)				
	HPV16+	HPV18+	12OT+	Negative	Total
HPV16+	**9**	0	0	1	10
HPV18+	0	**3**	0	0	3
12OT+	2	0	**22**	2	26
Negative	1	0	2	**68**	71
Total	12	3	24	71	**110** ^b^

*Note:* Kappa index: 0.89 (excellent with 94.4% concordance).

^a^ hr‐HPV test (*Cobas HPV test*; Roche Diagnostics): detection and genotyping HPV16, 18 and 12 others (12OT) pooled high‐risk HPV.

^b^ One test had a positive result for HPV16 and 18, and both results were considered separately.

The three discordant negative cases in each PM were not from the same women, although overall, they presented the same final cytological diagnoses: two cases with HPV12OT+/negative cytology for each medium, one case with HPV16+/LSIL in the candidate PM, and one case with HPV16+/negative in the reference PM.

In the analysis of test‐to‐test result agreement, by individual case, the agreement by medium remained excellent, with 92.7% (102/110) and a Kappa index of 0.86.

Considering the samples with satisfactory DNA extraction, the performance of the HPV tests across preservation mediums was nearly identical, with a 34% positivity rate (38/111 for the candidate medium and 38/109 for the reference medium). Based on the final diagnosis of CIN2+ at the time of sample collection, the sensitivity was 92.9% for both mediums (specificity = 74%, negative predictive value = 99% and positive predictive value = 34%).

For the cytology analysis, 96 paired cases by medium were considered, with the slide preparations processed together, balancing the time interval between sample collection and processing. The cytological smears characteristics and the diagnoses by medium and by LBC processing system are shown in Table 2.

**TABLE 2:** Cytological smears characteristics and the diagnoses from 96 women according to the liquid preservation medium (PM) and processing system (P) for liquid‐based cytology.

Cytology^a^	Processor for liquid‐based cytology (*n*)					
	P‐Cytoliq			P‐Thinprep		
	Candidate PM	Reference PM	*p* ^b^	Candidate PM	Reference PM	*p* ^b^
Parameters						
**Cellularity**						
Satisfactory	92	85	0.059	85	85	> 0.99
Unsatisfactory	4	11		11	11	
**SCJ component**						
Yes	56	51	0.468	49	39	0.148
No	40	45		47	57	
**Obscuring factor**						
Yes	23	15	0.147	11	16	0.299
No	73	81		85	80	
**Adequacy**						
Yes	92	87	0.125	82	86	0.650
No	4	9		14	10	
**Diagnoses**						
Negative	80	77	0.489	70	73	0.736
ASC‐US	6	4		6	1	
LSIL	2	4		7	7	
ASC‐H	3	1		2	1	
HSIL	1	1		1	3	
Unsatisfactory	4	9		10	11	

Abbreviations: ASC‐H, atypical squamous cells, cannot exclude HSIL; ASC‐US, atypical squamous cells of undetermined significance; LSIL‐HSIL, low‐grade or high‐grade squamous intraepithelial lesions; SCJ, squamocolumnar cell junction.

^a^ Parameters and diagnoses according to the Bethesda System [21, 22].

^b^ Chi‐square or Fisher's exact tests; statistics considered ASC‐US plus LSIL and ASC‐H plus HSIL.

**FIGURE 1:**
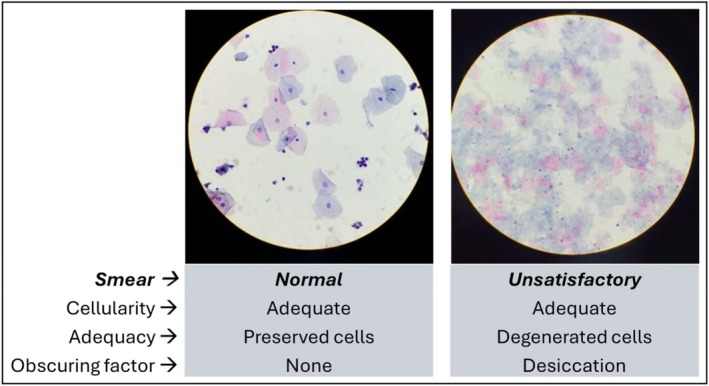
Liquid‐based cytology smears produced from samples preserved in the candidate medium and Cytoliq slide processor (both were first collected samples).

Considering the LBC smear produced by each of the four possible combinations between the two PM and the two LBC processors, the parameters analysis of the cellularity, presence of the SCJ component, obscuring factors and adequacy showed no significant differences (Table 2, Figure 1). When comparing the two PMs on the same LBC processor, no statistical difference was observed regarding cytological smear characteristics and the diagnoses (Table 2). When comparing the cytological diagnoses by PM‐Processor system from the same manufacturer, no significant difference was found, with the same number of diagnoses of ASC‐US/LSIL (*n* = 8) and ASC‐H/HSIL (*n* = 4) for each (*p* > 0.99; Table 2).

In a detailed analysis of unsatisfactory LBC cases, only one out of four cases on the candidate PM‐Cytoliq processor system and the 11 cases on the reference PM‐ThinPrep processor system had identical results. The four unsatisfactory cases in the candidate PM‐Cytoliq processor system had negative results for the LBCs from the ThinPrep processor. Among the 11 unsatisfactory cases in the reference homologous system, the LBCs produced on the Cytoliq processor had seven negative diagnoses, one ASC‐US, and two ASC‐H.

The agreement between diagnoses in LBC by medium, excluding unsatisfactory samples, was 94.0% (78/83) with a Kappa index of 0.79 (‘substantial’) when produced on the ThinPrep processor, and 91.8% (78/85) with a Kappa index of 0.63 (‘substantial’) when produced on the Cytoliq processor (Table 3).

**TABLE 3:** Agreement between cytological diagnoses (*n*) by liquid preservation medium (PM) and processing system for liquid‐based cytology.

Diagnostics in the reference PM	Processor for liquid‐based cytology (*n*)									
	Cytoliq					ThinPrep				
	Diagnostics in the candidate PM					Diagnostics in the candidate PM				
	Neg	ASC‐US/LSIL	ASC‐H/HSIL	Unsat	Total	Neg	ASC‐US/LSIL	ASC‐H/HSIL	Unsat	Total
Neg	**71**	2	2	2	77	**67**	4	—	2	73
ASC‐US/LSIL	3	**5**	—	—	8	—	**8**	—	—	8
ASC‐H/HSIL	—	—	**2**	—	2	1	—	**3**	—	4
Unsat	5	1	1	**2**	9	2	1	—	**8**	11
Total	79	8	5	4	**96**	70	13	3	10	**96**

*Note:* Kappa index for slide processor Cytoliq: 0.63 (91.8% concordance); Kappa index for slide processor ThinPrep: 0.79 (94.0% concordance).

Abbreviations: ASC‐H, atypical squamous cells, cannot exclude HSIL; ASC‐US, atypical squamous cells of undetermined significance; LSIL‐HSIL, low‐grade or high‐grade squamous intraepithelial lesions; Neg, negative; Unsat, unsatisfactory [22].

## 4 Discussion

The PM candidate demonstrated non‐inferior performance to the reference in detecting DNA‐HPV and producing LBC smears based on comparing results from cervical samples collected from the same women. For DNA‐HPV tests, there was only one insufficient sample for the PM candidate, and the agreement between the HPV test results, including genotyping, was high (> 92%), both for the total in each group and in the paired, case‐by‐case comparison, with an excellent Kappa index (> 0.86). Considering the final diagnosis of CIN2+, the sensitivity achieved by the hr‐HPV test was high at 92.9% for both PM. For LBC, the qualitative analysis of the smears produced and the cytological diagnoses showed no differences between the four combinations of PM‐Processors of slides evaluated.

Cervicovaginal sample preservation media are a crucial part of cervical pre‐cancer screening in HPV‐based testing programmes, and they must have the ability to preserve samples for several weeks to perform biomolecular tests such as DNA detection, mRNA or DNA methylation, as well as for cytological evaluation, meaning they need to preserve both cellular morphology and nucleic acids. Additionally, the PM should not cause false‐negative results.

In our evaluation, the candidate PM demonstrated robust results for screening tests and excellent agreement with the reference PM results, both achieving a sensitivity of 92.9%. We found the same 34.9% rate of hr‐HPV detection in samples preserved in both PMs, along with high agreement rates in the results, whether for hr‐HPV positivity, viral genotyping or LBC diagnoses. The negative predictive value of 98.6% for both PMs indicates safety for population‐based screening programmes.

For this clinical performance analysis of the candidate PM in cervical screening cytology, the steps followed adhered to a validation guide for new HPV tests established and used for over a decade [19], and the 90% sensitivity reference and high agreement rates (Kappa index > 0.70) were achieved by the candidate PM in this study. The performance for producing LBC smears was evaluated according to Bethesda System quality criteria [21, 22]. It was not inferior to that of samples from the same women preserved in the reference PM. Therefore, the PM candidate demonstrated clinical performance that justifies continuing the validation process for its use in cervical cancer screening.

A detailed evaluation of the unsatisfactory LBC cases highlighted that there were four cases in the same trade candidate PM‐Cytoliq processor system (Erviegas), all with negative results in the homologous reference system, compared to 11 unsatisfactory LBC cases in the reference same trade system, with the ThinPrep processor (Hologic), which the evaluation by the candidate homologous system showed seven negative diagnoses, one ASC‐US and two ASC‐H for the same women. Overall, the few observed discrepancies, although not significant, tended to favour the candidate system, and interchangeability between the systems showed equivalent results.

Some limitations of the study can be considered, as the sequential sample collection follows an initial routine sample collection, which could lead to lower cellular sampling in the last collection. However, this was possibly controlled by randomising the order of collections. Additionally, the time elapsed between collection and processing for LBC was not considered, although it was similar for the 96 paired cases processed simultaneously, ranging from 67 to 147 days. The study's strengths include the standardisation of procedures, all performed by trained professionals in a university environment, including reference laboratories and cytologists with experience in routine and large‐scale testing. Furthermore, the sample collections were done simultaneously, from the same woman, and in a randomised order for the first collection stored in both PMs. Among the cases studied, there was a high proportion of risk cases for positive HPV testing, with 34.9% detection, highlighting the high sensitivity found for both PM. The HPV test and reference LBC processor used in this study are among the most widely used in routine practice globally.

## 5 Conclusion

This study demonstrated that the PM candidate effectively preserved cervical samples for DNA‐HPV detection and LBC, being non‐inferior to the reference, supporting its use in cervical cancer screening and expanding the supply alternatives for the expected expansion of molecular biology‐based programmes. Future monitoring of the candidate PM's use on a larger scale may reveal additional particularities or further adjustments.

## Author Contributions

J.C.T., D.B.V., M.G.D., L.D.A. and A.G.S. developed the research protocol. J.C.T. coordinated all study phases and manuscript writing. D.B.V., M.G.D., L.D.A. and A.Y. collected the samples. M.G.D., L.D.A. and A.G.S. coordinated and executed the laboratory procedures. J.C.T., D.B.V., M.G.D. and L.D.A. collected and analysed the data. J.C.T., M.G.D. and L.D.A. drafted the manuscript. All authors reviewed the manuscript.

## Disclosure

Some parts of this manuscript were derived from an academic master's dissertation by L Assunção under the tutorial of J Teixeira and M Discacciati submitted to the Postgraduate Program of Obstetrics and Gynecology from the University of Campinas, Brazil.

## Conflicts of Interest

The authors declare no conflicts of interest.

## Data Availability

The data that support the findings of this study are available from the corresponding author upon reasonable request.

## References

[1] D. Singh , J. Vignat , V. Lorenzoni , et al., “Global Estimates of Incidence and Mortality of Cervical Cancer in 2020: A Baseline Analysis of the WHO Global Cervical Cancer Elimination Initiative,” Lancet Global Health 11, no. 2 (2023): 197–206. 10.1016/S2214-109X(22)00501-0 PMC9848409 36528031

[2] Estimate/2023 ‐ Cancer Statistics (Instituto Nacional de Cancer Jose Alencar Gomes da Silva (INCA), 2023), https://www.gov.br/inca/pt‐br/assuntos/cancer/numeros.

[3] V. Bouvard , N. Wentzensen , A. Mackie , et al., “The IARC Perspective on Cervical Cancer Screening,” New England Journal of Medicine 385, no. 20 (2021): 1908–1918. 34758259 10.1056/NEJMsr2030640 PMC12125667

[4] G. Ronco , J. Dillner , K. M. Elfström , et al., “Efficacy of HPV‐Based Screening for Prevention of Invasive Cervical Cancer: Follow‐Up of Four European Randomized Controlled Trials,” Lancet 383, no. 9916 (2014): 524–532, 10.1016/S0140-6736(13)62218-7. 24192252

[5] C. Gilham , A. Sargent , H. C. Kitchener , and J. Peto , “HPV Testing Compared With Routine Cytology in Cervical Screening: Long‐Term Follow‐Up of ARTISTIC RCT,” Health Technology Assessment 23, no. 28 (2019): 1–44. 10.3310/hta23280 PMC6600121 31219027

[6] R. B. Perkins , N. Wentzensen , R. S. Guido , and M. Schiffman , “Cervical Cancer Screening: A Review,” JAMA 330 (2023): 547–558. 37552298 10.1001/jama.2023.13174

[7] T. C. Wright , M. H. Stoler , C. M. Behrens , A. Sharma , G. Zhang , and T. L. Wright , “Primary Cervical Cancer Screening With Human Papillomavirus: End of Study Results From the ATHENA Study Using HPV as the First‐Line Screening Test,” Gynecologic Oncology 136 (2025): 189–197. 10.1016/j.ygyno.2014.11.076 25579108

[8] F. Inturrisi , J. A. Bogaards , D. A. M. Heideman , C. J. L. M. Meijer , and J. Berkhof , “Risk of Cervical Intraepithelial Neoplasia Grade 3 or Worse in HPV‐Positive Women With Normal Cytology and Five‐Year Type Concordance: A Randomized Comparison,” Cancer Epidemiology, Biomarkers & Prevention 30, no. 3 (2021): 485–491. 10.1158/1055-9965.EPI-20-1336 33293342

[9] M. Rebolj , C. S. Mathews , F. Pesola , et al., “Age‐Specific Outcomes From the First Round of HPV Screening in Unvaccinated Women: Observational Study From the English Cervical Screening Pilot,” BJOG 129, no. 8 (2022): 1278–1288, 10.1111/1471-0528.17058. 34913243

[10] J. C. Teixeira , D. B. Vale , C. S. Campos , et al., “Transition From Opportunistic Cytological to Organized Screening Program With DNA‐HPV Testing Detected Prevalent Cervical Cancers 10 Years in Advance,” Scientific Reports 14, no. 1 (2024): 20761. 39237756 10.1038/s41598-024-71735-2 PMC11377760

[11] M. A. Smith , M. Sherrah , F. Sultana , et al., “National Experience in the First Two Years of Primary Human Papillomavirus (HPV) Cervical Screening in an HPV Vaccinated Population in Australia: Observational Study,” BMJ 30, no. 376 (2022): e068582. 10.1136/bmj-2021-068582 PMC8965648 35354610

[12] A. C. Chrysostomou , D. C. Stylianou , A. Constantinidou , and L. G. Kostrikis , “Cervical Cancer Screening Programs in Europe: The Transition Towards HPV Vaccination and Population‐Based HPV Testing,” Viruses 10 (2018): 729. 30572620 10.3390/v10120729 PMC6315375

[13] F. F. Hamers , A. I. Poullié , and M. Arbyn , “Updated Evidence‐Based Recommendations for Cervical Cancer Screening in France,” European Journal of Cancer Prevention 31, no. 3 (2022): 279–286. 34191754 10.1097/CEJ.0000000000000701 PMC8974187

[14] M. Rebolj , C. S. Mathews , K. Denton , and HPV Pilot Steering Group , “Cytology Interpretation After a Change to HPV Testing in Primary Cervical Screening: Observational Study From the English Pilot,” Cancer Cytopathology 130, no. 7 (2022): 531–541. 35377967 10.1002/cncy.22572 PMC9542289

[15] I. A. Park , S. N. Lee , S. W. Chae , K. H. Park , J. W. Kim , and H. P. Lee , “Comparing the Accuracy of ThinPrep Pap Tests and Conventional Papanicolaou Smears on the Basis of the Histologic Diagnosis:A Clinical Study of Women With Cervical Abnormalities,” Acta Cytologica 45 (2001): 525–531. 11480713 10.1159/000327859

[16] R. S. Hoda , K. Loukeris , and F. W. Abdul‐Karim , “Gynecologic Cytology on Conventional and Liquid‐Based Preparations: A Comprehensive Review of Similarities and Differences,” Diagnostic Cytopathology 41 (2013): 257–278. 22508662 10.1002/dc.22842

[17] CONITEC, Comissão Nacional de Incorporação de Tecnologias no Sistema Unico de Saude, Brasil , “PORTARIA SECTICS‐MS N° 3: Decision to Incorporate Molecular Tests for the Detection of Oncogenic HPV Within the Scope of the ‘Unified Health System’ – ‘SUS’,” (2024), https://www.gov.br/conitec/pt‐br/midias/relatorios/portaria/2024/portaria‐sectics‐ms‐no‐3‐de‐7‐de‐marco‐de‐2024/view.

[18] ANVISA , “Agencia Nacional de Vigilancia Sanitaria,” (2024), Brazil, Health Products, CytoLiq ‐ Liquid‐Based Fixing Solution, Register No. 10039370007, https://consultas.anvisa.gov.br/#/saude/25351662189202111/?cnpj=46271011000107&situacaoNotificacaoRegistro=1.

[19] C. J. Meijer , J. Berkhof , P. E. Castle , et al., “Guidelines for Human Papillomavirus DNA Test Requirements for Primary Cervical Cancer Screening in Women 30 Years and Older,” International Journal of Cancer 124 (2009): 516–520. 18973271 10.1002/ijc.24010 PMC2789446

[20] J. R. Landis and G. G. Koch , “The Measurement of Observer Agreement for Categorical Data,” Biometrics 33, no. 1 (1977): 159–174. 843571

[21] G. G. Birdsong and D. D. Davey , “Specimen Adequacy,” in The Bethesda System for Reporting Cervical Cytology: Definitions, Criteria, and Explanatory Notes, 3rd ed., ed. R. Nayar and D. C. Wilbur (Springer, 2015).

[22] M. A. Pangarkar , “The Bethesda System for Reporting Cervical Cytology,” CytoJournal 19, no. 30 (2022): 19–28. 35673697 10.25259/CMAS_03_07_2021 PMC9168399

